# Uncovering the Molecular Basis for the Better Gefitinib Sensitivity of EGFR with Complex Mutations over Single Rare Mutation: Insights from Molecular Simulations

**DOI:** 10.3390/molecules27123844

**Published:** 2022-06-15

**Authors:** Miaomiao Li, Mengrong Li, Yanjie Xie, Jingjing Guo

**Affiliations:** 1 College of Life Sciences, Nanjing Agricultural University, Nanjing 210095, China; 2021216040@stu.njau.edu.cn (M.L.); 2019216037@njau.edu.cn (M.L.); yjxie@njau.edu.cn (Y.X.); 2 Engineering Research Centre of Applied Technology on Machine Translation and Artificial Intelligence, Faculty of Applied Science, Macao Polytechnic University, Macao 999078, China

**Keywords:** EGFR rare mutants, EGFR complex mutations, molecular dynamics simulation, steered molecular dynamics simulation, Gefitinib (IRE)

## Abstract

Epidermal growth factor receptor (EGFR) is an intensively focused target for anti-tumor compounds used in non-small cell lung cancer (NSCLC) therapy. Compared to the classical activating mutations, there are still many uncommon EGFR mutations associated with poorer responses to EGFR inhibitors. A detailed understanding of the molecular basis for multiple EGFR mutants exhibiting diverse responses to inhibitors is of critical importance for related drug development. Herein, we explored the molecular determinants contributing to the distinct responses of EGFR with a single rare mutation (G719S) or combined mutations (G719S/L858R and G719S/l861Q) to Gefitinib (IRE). Our results indicated that interactions, formed within the tetrad of residues S768 (in the αC-helix), D770 (in the αC-β4 loop), Y827 (in the αE-helix), and R831 (in the catalytic loop), play an important role in the stability of αC-helix and the maintenance of K745–E762 salt bridge in the absence of IRE, which are weakened in the EGFR^G719S^ system and enhanced in the EGFR^G719S/L858R^ system upon IRE binding. Besides, the introduced hydrogen bonds by the co-occurring mutation partner also contribute to the stability of αC-helix. The work done for inhibitor dissociation suggests that IRE exhibits a stronger binding affinity to EGFR^G719S/L858R^ mutant. Together, these findings provide a deeper understanding of minor mutations, which is essential for drug development targeting EGFR with less common mutations.

## 1. Introduction

Many human cancers’ occurrence is associated with the abnormal activation of tyrosine kinase [1,2]. Abundant genomic alterations in epidermal growth factor receptor (EGFR) have been revealed, including point mutations, deletions, and insertions within exon 18–21 of the EGFR gene [3,4,5]. The mutational constitutive activation of the kinase domain of EGFR is linked to non-small cell lung cancer (NSCLC), [6,7,8] accounting for 80% of lung cancer [9]. About 85% of observed EGFR mutations are common mutations occurring frequently in NSCLC patients, while the remaining 15% cover various rare mutations [3,10]. To treat lung cancer, characterized as the leading cause of morbidity and mortality, some targeted drugs targeting EGFR have been developed and approved, such as Gefitinib (IRE), Erlotinib, and Afatinib [11,12,13,14]. Gefitinib has a novel antitumor effect on NSCLC and has been documented to be useful in patients previously treated with chemotherapy regimens [15], or in selected groups of patients as first-line therapy, with effects of tumor regression and symptom amelioration [16].

Among rare EGFR mutations, G719X (including G719S, G719A, G719C, and G719D) substitutions in the phosphate-binding “P-loop” within the N-lobe, is one of the most commonly observed mutations, and represent roughly 1.5–3% of all EGFR mutations in NSCLC patients [10] G719X mutations could occur as an independent EGFR one or together with other additional point mutations (rare or classical) as compound EGFR mutations [17]. Complex mutations have been reported to account for about 5–15% of EGFR mutations. [18,19,20,21] Effective response to IRE is associated with EGFR mutations, as reported in various studies. Chiu et al. [22] assessed the effectiveness of IRE in patients with rare G719X mutation, common L858R mutation, and rare–rare EGFR mutation combinations. The clinical study focused on uncommon EGFR mutations revealed that although patients with a single G719X mutation were sensitive to IRE treatment, they were markedly less sensitive compared to those with the classical L858R mutation. Furthermore, the results of rare and rare combined mutations are heterogeneous as patients with G719X + L861Q exhibit more impressive outcomes than those with G719X + S768I. Hence, it is likely that the sensitivity of NSCLC patients to EGFR inhibitors is influenced by the specific co-occurring mutation partner within rare and rare combinations [22]. Beau-Faller et al. and Cheng et al. also uncovered a better response to IRE for EGFR with complex mutations than a single rare mutation [17,23]. EGFR featured with complex mutations exhibits a stronger sensitivity to IRE, especially under a rare mutation combined with a classical mutation excluding the T790M mutation [23,24].

NSCLC-diagnosed patients exhibit diverse outcomes upon IRE treatment based on clinical study, yet molecular determinants behind the different performances are not fully understood. Many studies have focused on common mutations such as L858R and T790M, [25,26] but less is known for identifying rare mutations at the molecular level. In view of the high incidence of lung cancer, there are a lot of NSCLC-diagnosed patients found to harbor rare EGFR mutations [27]. Therefore, a detailed understanding of minor mutations is favorable to assess the current treatment options and develop new effective therapeutic strategies [28].

For this purpose, we carried out a systematic computational study to provide insights into different performances of IRE on multiple EGFR mutants. In the present work, conventional molecular dynamic (CMD) simulations and steered molecular dynamic (SMD) simulations have been carried out to explore the characteristics of EGFR mutants. A total of six systems were considered, including APO and IRE-bound EGFR mutants: rare (G719S), rare + rare (G719S/L861Q), and rare + classical (G719S/L858R). Our analyses showed that in the absence of IRE, cancer mutations favor the active αC-in conformation, and the stability of αC-helix is mediated by hydrogen bond interactions formed within a tetrad of residues S768 (in the αC-helix), D770 (in the αC-β4 loop), Y827 (in the αE-helix), and R831 (in the catalytic loop). Upon IRE binding, the favorable interactions were disrupted in the EGFR^G719S^ system, whereas enhanced in the EGFR^G719S/L858R^ system. Besides, the free energy profile suggests that IRE has a stronger binding affinity to EGFR^G719S/L858R^ mutant. These results provide us with serviceable insights on the better IRE sensitivity of the EGFR compound mutant over the single rare one, which is useful for developing effective therapeutic strategies.

## 2. Results and Discussion

### 2.1. EGFR Minor Mutations Favor the Active αC-in Conformation

**Figure 1:**
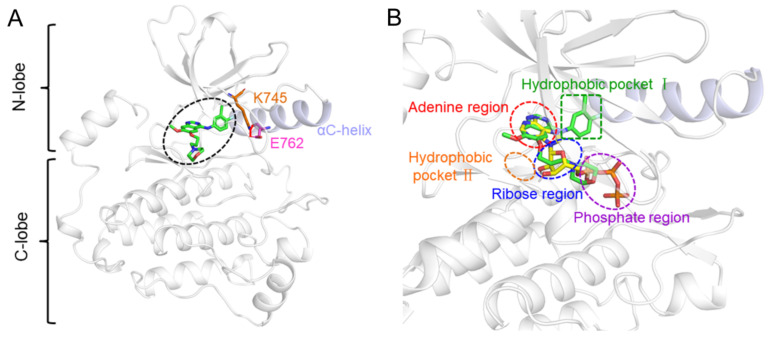
The structure of EGFR is complex with IRE in the absence (**A**) and presence (**B**) of ATP. The αC-helix is marked in light blue. The residues E762 and K745 forming a salt bridge are shown as orange and magenta sticks, respectively. The small molecules IRE and ATP are shown as green and yellow sticks, respectively. The IRE binding site is highlighted by a black circle in (**A**), and the five regions consisting of the catalytic cleft are highlighted by circles with different colors in (**B**).

EGFR is a transmembrane receptor tyrosine kinase, of which the kinase domain comprises two lobes (Figure 1A), namely the N-terminal and the C-terminal lobes. The catalytic cleft formed between the two lobes consists of two distinct binding pockets. The one in the front is the ATP-binding site, while the back one is a non-ATP-contact region. The general kinase ATP-binding pocket could be divided into four regions: the adenine region, the ribose region, the phosphate region, and the hydrophobic pocket II. In the active conformation, the non-ATP-contact region is primarily the hydrophobic pocket I, which also is identified as the affinity and selectivity pocket, and hence is important for inhibitor binding [29]. Apart from the orthosteric site occupied by the majority of inhibitor IRE, the benzene ring part is extended to the hydrophobic pocket I (Figure 1B). Moreover, for the active EGFR conformation, the αC-helix is positioned in an “in” conformation and the K745–E762 salt bridge is present.

The root mean square deviation (RMSD) of the backbone atoms is generally considered to be an indicator of stability. Firstly, the RMSDs of the whole EGFR kinase domain in distinct forms were calculated according to their initial positions to describe the conformational stability of protein. As presented in Figure S1, the RMSDs of most simulations are stable and smaller than 3 Å on the whole, indicating the high stability of EGFR systems during the course of simulations; therefore, the studied systems performed three parallel runs of 800 nanoseconds is feasible [30]. The equilibrium periods of the trajectories were determined to be between 600 and 800 ns in unbound and IRE-bound EGFRs (Figure S1); therefore, the next analyses were based on the equilibrium portion of the trajectories. Then, to intuitively compare the stability of functional region αC-helix, we also performed a distribution calculation based on the RMSD values of αC-helix backbone atoms in the absence of IRE. It has been well established that the active αC-in conformation is characterized by the placement of the αC-helix to maintain the catalytically important salt bridge formed between K745 and E762 (referred to hereafter as KE salt bridge) [31]. Therefore, the distance between the charged groups of K745 and E762 was also calculated.

**Figure 2:**
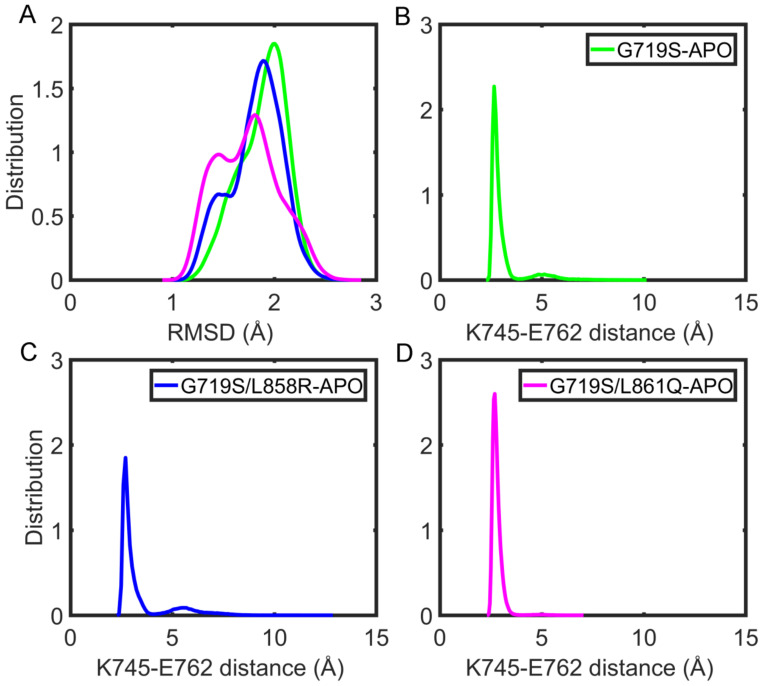
The stability of the αC-helix and KE salt bridge during the simulations for APO systems. (**A**) The RMSD values of the αC-helix backbone atoms in the last 200 ns MD trajectories using the initial structure as the reference. (**B**–**D**) The probability distribution profile of the distance between atoms K745/NZ and E762/(OE1, OE2) during the last 200 ns is based on all three replicates.

From the RMSD distribution figure, it is found that the αC-helix exhibits lower flexibility with the average RMSD values below 2 Å among the three APO systems (Figure 2A). Additionally, from the distance calculation, it is obtained that the average K745–E762 distances are 3.05 ± 0.83 Å for the G719S-APO system, 3.35 ± 1.12 Å for the G719S/L858R-APO one, and 2.79 ± 0.27 Å for the G719S/L861Q-APO one, respectively. The results indicate that compared with the single rare mutation, rare–rare combined mutations result in the K745–E762 distance value shifting to a relatively smaller area, while rare-classical combinations induce a broader distribution area of the K745–E762 distance values (Figure 2B–D). On the whole, such changes observed in the mutant systems indicate that EGFR mutants tend to sample the active αC-in conformation with the presence of the KE salt bridge.

### 2.2. Identification of Favorable Interactions Contributing to the Stability of the αC-Helix

In order to investigate the dynamic behaviors of EGFR mutants, the root-mean-square fluctuation (RMSF) of Cα atoms of protein residues and the cross-correlations of Cα atomic displacements were calculated based on the last 200-ns MD trajectories considering all three replicates together. The important regions of EGFR were highlighted in different colors shown in (Figure S2) to visualize the spatial position of the regions mentioned next on the protein. As RMSF results indicated (Figure S3), the flexibility of the P-loop was reduced upon complex formation. This structure acts as a flexible clamp that covers and anchors the non-transferable phosphates of ATP for the EGFR kinase domain [32,33]. When the activate site was occupied by the inhibitor IRE, the reduced flexibility of the P-loop may contribute to its stable binding. It was also noted that the A-loop exhibited the highest flexibility in the G719S-IRE system compared with the other two IRE-bound EGFRs with complex mutations, which might be associated with different IRE sensitivity of EGFR in distinct forms.

**Figure 3:**
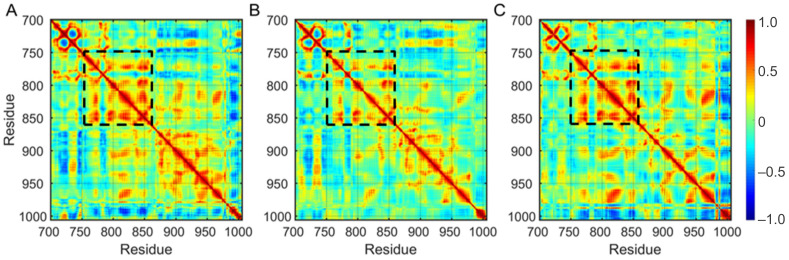
The covariance matrix maps of EGFR in the APO forms: (**A**) G719S-APO, (**B**) G719S/L858R-APO, and (**C**) G719S/L861Q-APO. The positively correlated regions existing in all APO systems are demonstrated by a black square box.

Through constructing a covariance matrix, the correlations of each pair of Cα atoms are intuitively clear. As presented in the covariance matrix maps (Figure 3), an apparent correlated motion is observed in the region spanning from K754 to L862, including the αC-helix, the αC-β4 loop, the hinge region, the αE-helix, the catalytic loop, and the N-terminal of the A-loop regions. These regions demonstrated by a black square box exhibit are positively correlated. Among these regions, the αC-β4 loop, the αE-helix, the catalytic loop, and the A-loop (N-terminal) regions are adjacent either to the αC-helix or to the hinge region which regulates the relative motions between the N lobe and the C lobe of kinase domain as well as the conformation of the binding site (Figure S2). The tetrad of residues S768 (in the αC-helix), D770 (in the αC-β4 loop), Y827 (in the αE-helix), and R831 (in the catalytic loop) make hydrogen bond interactions which, as a consequence, contribute to maintaining the αC-in conformation (Figure S4A).

In addition, hydrogen bonds, formed between the αC-helix and the β4-β5 loop or the A-loop, are also in favor of the stability of αC-in conformation as well as the KE salt bridge. The residue N756 in the αC-helix forms hydrogen bonds with S784, T785, and V786 on the β4-β5 loop (Figure S4B), and the residue E758 also located in the αC-helix interacts with R858, K860, and K875 on the A-loop (Figure S4B–D). Overall, the αC-in conformation was maintained during the simulations as a result of these formed favorable interactions.

### 2.3. IRE Binding Alters the Conformational Dynamics of EGFR Kinase Domain

**Figure 4:**
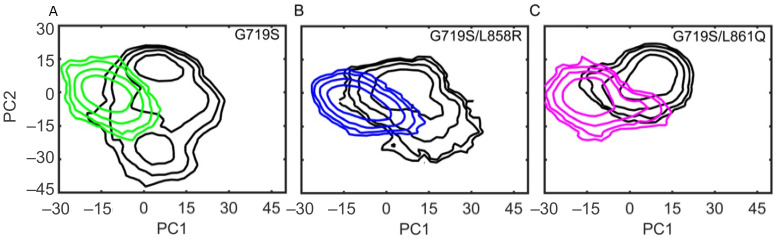
Conformational dynamics of EGFR in the APO and IRE-bound forms. The first two principal components (PCs) are shown in free energy surfaces. All APO forms are colored in black (**A**–**C**). Complex systems of G719S-IRE (**A**), G719S/L858R-IRE (**B**), and G719S/L861Q-IRE (**C**) are colored in green, blue, and magenta, respectively.

To characterize the conformational changes of the EGFR kinase domain upon IRE binding, principal component analysis was carried out on the last 200 ns MD trajectories considering three replicates together. The results are shown in the free energy surface using the first two principal components (named PC1 and PC2) (Figure 4). It is noted that for the EGFR^G719S^ mutant in APO form, the conformational space is wider along the PC2 direction, which is considered to be more flexible without the occupation of an inhibitor at the binding pocket (Figure 4A). Moreover, the G719S/L861Q-APO system is more stable demonstrated by the least conformational space (Figure 4C). All EGFR mutants in IRE-bound form discriminate from the APO one by PC1 (Figure 4A–C). Besides, the discrimination between the APO form and IRE-bound form is also according to PC2 for EGFR^G719S^ and EGFR^G719S/L858R^ mutants.

The flexibility of the EGFR kinase domain is reflected in a variation of the inter-lobe rearrangement. PC1 mainly captures the twisting motion of the N-lobe (Supplementary Movie S1), and PC2 illustrates an open-closed movement of the N-lobe as well as the rearrangement of the A-loop (Supplementary Movie S2), both resulting in the conformational change of binding site. Along the PC1 direction, the N-lobe as a whole presents an outward twisting motion in the IRE-bound form compared with in the APO one; thereby, as a consequence, the αC-helix has an outward displacement in the complex systems to enlarge the non-ATP contact region to fit the benzene ring part of IRE. Along the PC2 direction, the N-lobe exhibits a downward movement in the IRE-bound form compared with in the APO one, which makes the binding pocket more stable in the presence of IRE. Generally speaking, the inhibitor binding translates into protein conformational changes, which mainly affect the functionally important regions of the target protein. The binding pocket undergoes dynamical and conformational changes to better accommodate the inhibitor in the complex systems.

### 2.4. IRE Binding Destabilizes the αC-Helix and KE Salt Bridge in the G719S System

**Figure 5:**
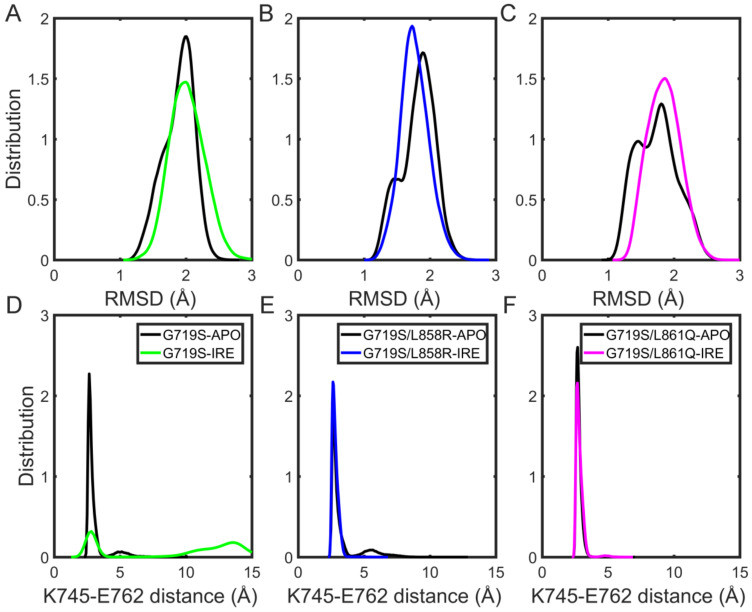
The stability of the αC-helix and KE salt bridge upon IRE binding during the simulations. The RMSD of the αC-helix backbone atoms in the absence and presence of IRE: (**A**) G719S mutants; (**B**) G719S/L858R mutants; (**C**) G719S/L861Q mutants. The probability distribution profiles of the distance between atoms K745/NZ and E762/(OE1, OE2) in the absence and presence of IRE: (**D**) G719S mutants with the APO form colored in black and with the complex form colored in green; (**E**) G719S/L858R mutants with the APO form colored in black and with the complex form colored in blue; (**F**) G719S/L861Q mutants with the APO form colored in black and with the complex form colored in magenta.

The αC-helix serves as an important functional region of EGFR, of which RMSD is also calculated to character its conformational stability in the IRE-bound form. As shown in Figure 5, the αC-helix is relatively more flexible in the IRE-bound G719S system with the largest RMSD value of 2.02 ± 0.27 Å (Figure 5A). For the G719S/L858R-IRE system, the binding of IRE reduces the flexibility of the αC-helix, demonstrated by the average RMSD value of 1.77 ± 0.22 Å in the IRE-bound form and 1.82 ± 0.26 Å in the APO one (Figure 5B). For the G719S/L861Q-IRE system, IRE binding also contributes to less flexibility of the αC-helix with a smaller RMSD value distribution area compared with that in the APO form (Figure 5C).

The KE salt bridge serves as a proxy for characterizing the αC-in conformation, of which stability is associated with that of the αC-helix, thus the distance calculation is performed for the two charged residues to reflect the effect of IRE binding. From Figure 5D, it is found that in the IRE-bound G719S system, the KE salt bridge is disrupted, as demonstrated by the larger distance distribution with an average of 9.35 ± 4.84 Å. For the other two EGFR mutants in the IRE-bound form, the KE salt bridge is well maintained, with an average distance of 2.81 ± 0.26 Å in the EGFR^G719S/L858R^ system and 2.84 ± 0.32 Å in the EGFR^G719S/L861Q^ one (Figure 5E,F). Overall, the G719S/L858R combined mutations enhanced the sensitivity for EGFR toward IRE based on the smallest RMSD value of αC-helix and KE distance value.

Shan et al. suggested the presence of a third conformation of EGFR kinase characterized by a broken KE salt bridge and partially unfolded αC-helix, which was an αC-out conformation that differed from the Src/CDK2-like (αC-out) inactive conformation [25]. It is worth mentioning that IRE is an ATP-competitive and reversible inhibitor, which binding to EGFR featured with an active αC-in conformation. For the G719S-IRE system, the K745–E762 distance change indicates that the αC-in conformation is less sampled during the simulation, and the αC-helix conformation might sample the above-mentioned third conformation, which might suggest less sensitivity of EGFR^G719S^ mutant to inhibitor IRE.

**Figure 6:**
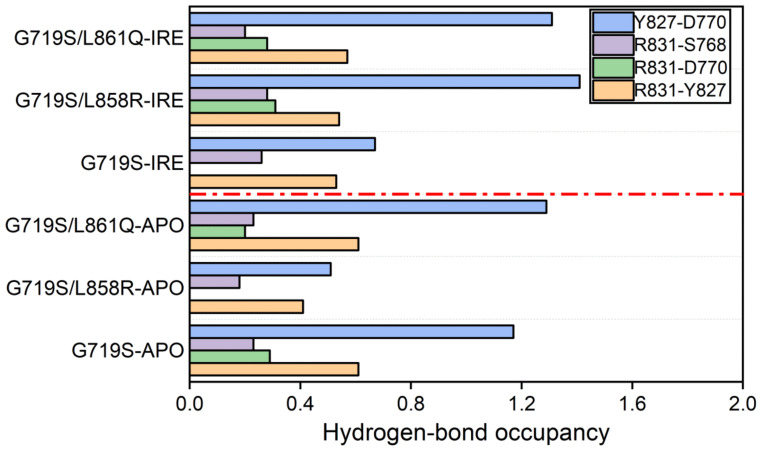
Hydrogen bond occupancy differs between the APO systems and the complex systems. Some residues have a hydrogen bond donor or acceptor of more than one, thus the hydrogen bond occupancy is above 1. A low panel of the red dotted line indicates the occupancy of hydrogen bonds (Y827-D770, R831-S768, R831-D770, and R831-Y827) in the APO systems, and the top panel indicates the occupancy of these hydrogen bonds in the complex systems.

By analyzing the hydrogen bond interactions mentioned above, it might provide a plausible explanation for the different observations for the αC-helix and KE salt bridge among the complex systems. Comparing the hydrogen bond occupancy between the APO systems and the complex ones (Figure 6), it is found that for the IRE-bound G719S system, the hydrogen bond formed between R831 and D770 disappears, and the occupancy of the Y827–D770 hydrogen bond is significantly reduced. For the G719S/L858R-IRE system, it is observed the formation of the R831–D770 hydrogen bond, and the prolonged existence of the Y827–D770 hydrogen-bond interaction. There is less change of hydrogen bond occupancy for the EGFR^G719S/L861Q^ mutant upon IRE binding. These hydrogen bond interactions could exert a favorable effect on the αC-in positioning. The broken and reduced interactions in the G719S-IRE system might give rise to less stability of the αC-helix.

Furthermore, the hydrogen bonds introduced by the co-occurring mutated residue also make a positive knock-on effect on the stability of αC-helix. For the G719S/L858R system, the hydrophobic leucine residue mutates to the charged arginine residue, and the latter makes interactions with other charged residues (R836 and D837) located at the catalytic loop (Figure S5A). For the G719S/L861Q system, the hydrophobic leucine residue mutates to the charged residue glutamine, similarly, the latter forms a hydrogen bond with R831 located at the catalytic loop and Y764 positioned at the αC-helix (Figure S5B,C). All these interactions are conducive to stabilizing the αC-helix indirectly. In words, it is reasonable to correlate the hydrogen bond interactions with the stability of αC-helix and, as observed in the G719S-IRE system, less stability of the αC-helix and broken KE salt bridge.

### 2.5. Effects of IRE Binding on the Binding Pocket

**Figure 7:**
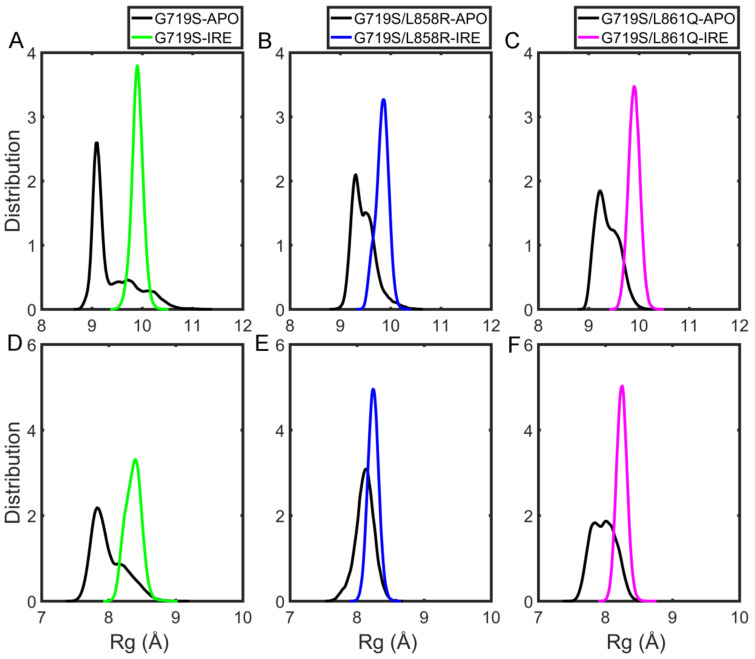
The probability distribution profiles of the *R*_g_ values of the binding site in the six systems considering three replicates together: (**A**) G719S mutants; (**B**) G719S/L858R mutants; (**C**) G719S/L861Q mutants. The probability distribution profiles of the *R*_g_ values of hydrophobic residues in the six systems considering three replicates together: (**D**) G719S mutants; (**E**) G719S/L858R mutants; (**F**) G719S/L861Q mutants.

To probe how IRE binding affects the binding pocket, the radius of gyration (*R*_g_) was calculated for residues consisting binding pocket during the simulations. *R*_g_ depends on the relationship between the mass of certain atoms and the center of gravity of the molecule, which could be used to characterize the compactness of the binding pocket here. As presented in Figure 7A–C, though the average *R*_g_ value is increased, it shows a smaller distribution area in the complex systems compared with the APO systems. It is suggested that the binding pocket is more dynamic over the course of the simulation in absence of IRE, and a relatively stable pocket is induced by IRE binding though it stretches out in the presence of IRE.

We then estimated the contact information between IRE and EGFR. As presented in Figure S6, the residues contacted with IRE are similar among the three complex systems, and frequent contact residues are mostly hydrophobic residues, such as L718, F723, V726, A743, I744, M766, L777, L788, I789, M790, L792, M793, P794, L799, and L844. Therefore, the hydrophobic interactions play an important role in the stabilization of IRE at the binding pocket. To further consider the compactness of the hydrophobic pocket, the *R*_g_ values were also calculated for the mentioned hydrophobic residues consisting of the binding site. As shown in Figure 7D–F, the *R*_g_ values distribute broadly in the APO systems, whereas IRE binding shifts the *R*_g_ distribution to a relatively smaller area with increased *R*_g_ values. These observations indicate that the hydrophobic pocket is relatively more compact but less stable in the absence of IRE, and upon IRE binding, the pocket enlarges slightly to better accommodate IRE and becomes more stable. Comparing the three complex systems, the *R*_g_ value of these hydrophobic residues in the G719S system is the biggest with an average of 8.37 ± 0.12 Å, whereas for the other two mutants, the average *R*_g_ values are similar with a smaller distribution area. In short, the results suggest that the compactness and stability of the hydrophobic cavity in the G719S-IRE system are weaker than the other two IRE-bound systems, which might affect the stability of IRE at the binding site.

**Figure 8:**
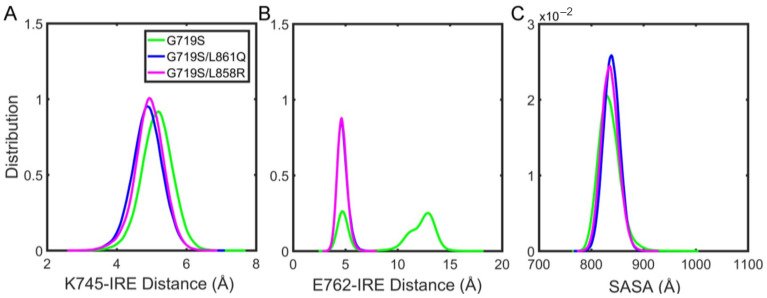
The probability distribution profiles of (**A**) the distance between atoms K745/NZ and IRE/F, (**B**) the distance between atoms E762/(OE1, OE2) and IRE/F, and (**C**) the solvent-accessible surface area (SASA) for the binding region of IRE benzene.

IRE majorly occupies the ATP-binding regions with the benzene ring together with halogen atom substituents embedded in the non-ATP-contact region (hydrophobic pocket I) (Figure 1B). The vicinity residues around the substituted fluorine atom contain K745 and E762. The fluorine atom has a strong negative charge, which could attract the positively charged K745, thus the stability of the benzene ring part at the pocket could be enhanced by the positive electrostatic interaction. The distance between the fluorine atom and polar nitrogen atom of K745 was calculated. From Figure 8A, the distance distribution area is relatively larger in the G719S-IRE system than that in the other two systems. In addition, the electronegativity of the fluorine atom also makes an effect on the negatively charged E762. Similarly, the distance between the fluorine atom and polar oxygen atoms of E762 was calculated (Figure 8B). For the G719S-IRE system, E762 is significantly distant from the fluorine atom, suggesting that the repulsion exerted by the fluorine atom on E762 is larger than that in other systems, which results in E762 moving to the outside of the binding pocket, thereby disrupting the KE salt bridge. Such a change might expand the solvent-exposed hydrophobic surface, and then weaken the stability of hydrophobic pocket I. Therefore, we quantified the area of the benzene ring part located exposed to the solvent through the solvent-accessible surface area (SASA) calculation. It is observed that obvious overlaps for SASA value distribution exist among the three complex systems, but the value is recognized to fluctuate to a larger area in the G719S-IRE system than in other systems. The observation suggested that the hydrophobic environment for the benzene ring binding is not stable as the one in other complex systems (Figure 8C), which might contribute less affinity of IRE toward EGFR^G719S^ mutant than other EGFR mutants with rare–rare and rare–classical combination.

### 2.6. Stronger Binding Affinity of IRE with EGFR^G719S/L858R^ Mutant

**Figure 9:**
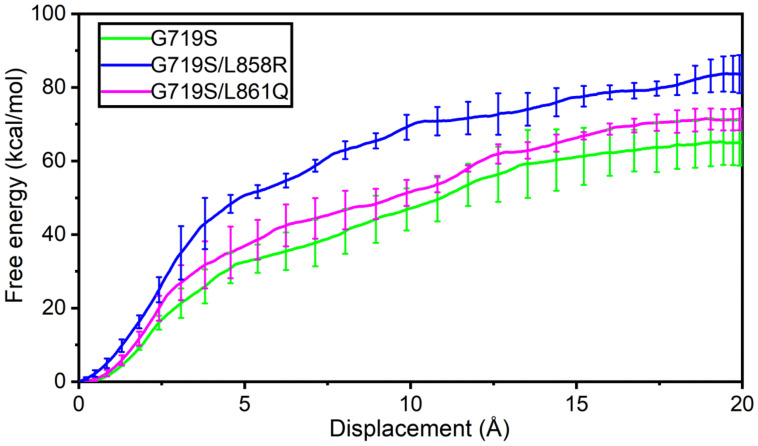
The average work profile for IRE dissociation with error bars of each complex system during the SMD simulations. The bars represent the standard deviation of the external works.

Steered molecular dynamics (SMD) simulation is an approximate and quicker approach for simulating ligand dissociation by applying an external force. To explore the binding affinity of IRE toward EGFR with various mutations, SMD simulations were performed to pull IRE out of the binding site, which could provide physical quantity, such as free energy profile change along the chosen reaction coordinate. For the competitive inhibitor IRE, its dissociation from distinct EGFR mutants is closely related to the inhibitory activity. As seen in Figure 9, pulling IRE out of EGFR^G719S/L858R^ mutant needs the most work (roughly 83 kcal/mol) to be done, while they work in the G719S/L861Q system is a bit higher than the one in the G719S system. In short, the free energy change difference reveals that IRE has a stronger binding affinity with EGFR harboring combined mutations than a single rare mutation alone. Additionally, a rare mutation combined with a classical L858R mutation may be a strong indicator of EGFR sensitivity to EGFR inhibitors.

## 3. Conclusions

This study provides insights into the molecular basis by which EGFR with complex mutations shows better sensitivity for IRE than with a single rare mutation. In particular, the EGFR^G719S-L858R^ mutant exhibits the strongest sensitivity for IRE among the simulated systems, which suggests that a rare EGFR mutation combined with a classical L858R mutation may be a strong indicator of sensitivity to EGFR inhibitor. In the absence of IRE, the computational structural analyses uncovered that minor mutations favor the αC-in conformation, and the hydrogen bonds formed within the tetrad of residues S768 (in the αC-helix), D770 (in the αC-β4 loop), Y827 (in the αE-helix), and R831 (in the catalytic loop) play an essential role for the stability of αC-helix and the maintenance of KE salt bridge. In the presence of IRE, the αC-helix is relatively more flexible for the EGFR^G719S^ mutant and the KE salt bridge is broken. The important interactions contributing to the stability of αC-helix are weakened in the IRE-bound EGFR^G719S^ system, whereas are enhanced in the IRE-bound EGFR^G719S-L858R^ system. The hydrogen bonds introduced by the second mutated residue also contribute to the stability of αC-helix. Moreover, the free energy change profiles reveal that the most work is needed to pull IRE out of the binding pocket in the EGFR^G719S/L858R^ system, and a bit above work is required to dissociate IRE in the EGFR^G719S/L61Q^ system than in the EGFR^G719S^ system, indicating that better affinity of IRE toward EGFR with complex mutations than single rare mutation. On balance, combined mutations show stronger sensitivity to inhibitor than the single minor mutation. An explicit understanding of rare mutations could provide useful insights for molecular drug design targeting EGFR uncommon mutations.

## 4. Methods

### 4.1. System Setup

The structures of mutated-EGFR in APO and IRE-bound forms were prepared based on the crystal structure of EGFR^G719S/T790M^ mutant in the complex of IRE (PDB ID: 3UG2 [34]). The completion of missing residues on the P-loop and α-loop was based on the template structure (PDB ID: 2ITY [35]). Moreover, the construction of the remaining missing amino acid residues in the crystal structure was achieved using the MODELLER [36] plugin in Chimera [37]. Mutated residues substitution and the removal of small molecules both were carried out by PYMOL [38]. The full names and abbreviations of amino acids and other nouns in this work are listed in Tables S1 and S2.

### 4.2. Conventional Molecular Dynamics (CMD) Simulation

All prepared structures were employed as the initial structures for the conventional MD simulations, and all simulations were performed in AMBER 18 [39]. The resp force field parameters for inhibitor IRE were generated by using Gaussian 09 combined with the Antechamber module in the Amber 18 package. The AMBER force fields ff14SB [40] and GAFF [41] were used for describing the protein molecule and the ligand respectively. All simulated systems were neutralized with appropriate numbers of Na^+^ ions. Each neutralized system was solvated in an orthogonal box with TIP3P water molecules [42], leaving at least 10Å between the solute atoms and the borders of the box. The particle mesh Ewald (PME) summation method [43] was applied to treat Coulombic interactions and the Shake algorithm [44] was used to constrain bond vibrations involving hydrogen atoms.

Three independent parallel molecular dynamics simulations were performed for each system. Four steps including the energy minimization, heating, equilibration, and production runs were carried out for the whole molecular simulation. Before MD simulations, each system was minimized for four 15,000 steps by means of the steepest descent and the conjugate gradient methods. The first three minimizations were performed with restraint force constants of 5.0, 2.0, and 1.0 kcal/mol/Å^2^ applied to all heavy atoms, the protein backbone atoms, and the Cα atoms, respectively. However, no restraint was imposed for the last minimization. Following the minimization steps, all simulated systems were heated from 0 to 298.15 K with a force constant of 5.0 kcal/mol/Å^2^ applied to all heavy atoms. Next, each system was equilibrated through two 1000-ps and one 2000-ps steps with force constants of 1.0, 0.5, and 0.1 kcal/mol/Å^2^ applied to all heavy atoms, the protein backbone atoms, and the Cα atoms, respectively. Finally, an 800-ns production run without any restraints was performed for all equilibrated systems at the isothermal-isobaric (NPT) ensemble using Berendsen barostat [45] with a target pressure of 1 bar. Coordinates of the production runs were recorded every 2.0 ps.

### 4.3. Steered Molecular Dynamics (SMD) Simulation

SMD is a technique to manipulate the system from an initial configuration to a target configuration by applying an external force [46,47,48]. In our work, the SMD simulations were performed by applying forces along the line connecting the center of mass (COM) of IRE and residues within 6 Å of IRE. The applied force along the identified direction accelerated the release of IRE out of the binding pocket of EGFR. The three independent 2-ns SMD simulations in all systems were started with the spring constant of 10 kcal/mol/Å^2^ controlling the strength of the pulling force. To avoid protein drift during the pulling process, the restriction was imposed on protein regions beyond 6 Å of IRE.

### 4.4. Principal Component Analysis

Principal component analysis (PCA) on EGFR in APO or inhibitor-bound forms based on the last 200-ns MD trajectories was performed using the CPPTRAJ module of AMBER18 software. The covariance matrix was firstly calculated using the coordinates of Cα atoms of EGFR by RMSD fitting and then diagonalized to obtain the principal component eigenvectors. Each selected conformation was then projected in the collective coordinate space defined by the two largest principal component eigenvectors.

### 4.5. Probability Density Profile

The density distribution profiles were calculated in Matlab employing the Ksdensity function.

## Data Availability

Molecular Dynamics trajectories and topology files of simulated systems in this work could be downloaded in https://zenodo.org/record/6616150#.YqWW6MjjxTK.

## Supplementary Materials

The following supporting information can be downloaded at: https://www.mdpi.com/article/10.3390/molecules27123844/s1. Figure S1: The RMSDs of unbound EGFRs and IRE-bound EGFRs as a function of simulation time. Figure S2: The structure of EGFR with important regions highlighted. Figure S3: The flexibility of EGFRs during the simulations. Figure S4: The hydrogen-bond interactions contribute to the stability of the αC-helix. Figure S5: The hydrogen-bond interactions introduced by the co-occurring mutated residue. Figure S6: Contact map between IRE and EGFR in the three IRE-bound systems. Table S1: The full names and abbreviations of amino acids in this work. Table S2: The full names and abbreviations of nouns in this work. Movie S1: The motion of EGFR captured in PC1. Movie S2: The motion of EGFR captured in PC2.
