# Supplementary material for: Uncovering the Molecular Basis for the Better Gefitinib Sensitivity of EGFR with Complex Mutations over Single Rare Mutation: Insights from Molecular Simulations
Source: Molecules. 2022 Jun 15;27(12):3844. doi: 10.3390/molecules27123844 (PMC9230809; doi:10.3390/molecules27123844)
Supplement: Supplementary file 1 [file molecules-27-03844-s001.zip › Rare mutation SI-new.pdf]

Uncovering the molecular basis for the better Gefitinib sensitivity of EGFR with complex mutations over single rare mutation: insights from molecular simulations

Miaomiao Li[1], Mengrong Li[1], Yanjie Xie[1], Jingjing Guo[1,2*]
[1]College of Life Sciences, Nanjing Agricultural University, Nanjing 210095, China
[2]Engineering Research Centre of Applied Technology on Machine Translation and Artificial Intelligence, Faculty of Applied Science, Macao Polytechnic University, Macao 999078, China
[*] Corresponding author: guojj@njau.edu.cn

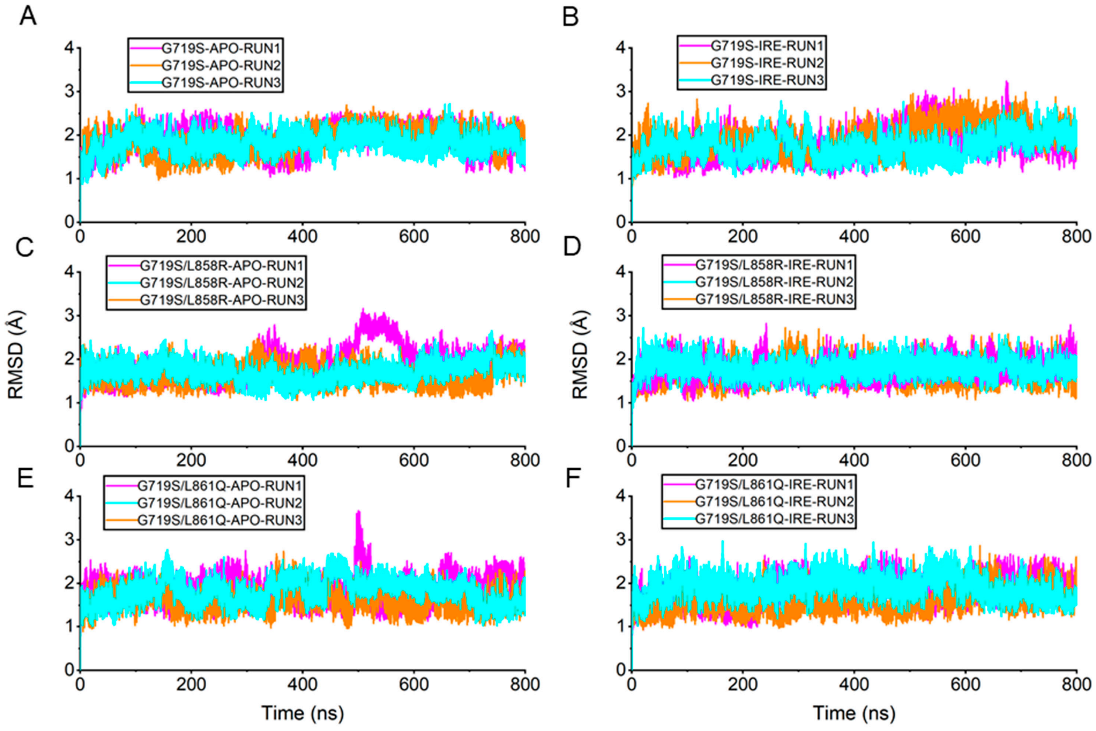

Figure S1. The RMSDs of unbound EGFRs and IRE-bound EGFRs as a function of simulation time. (A) G719S-APO; (B) G719S-IRE; (C) G719S/L858R-APO; (D) G719S/L858R-IRE; (E) G719S/L861Q-APO; (F)G719S/L861Q-IRE.

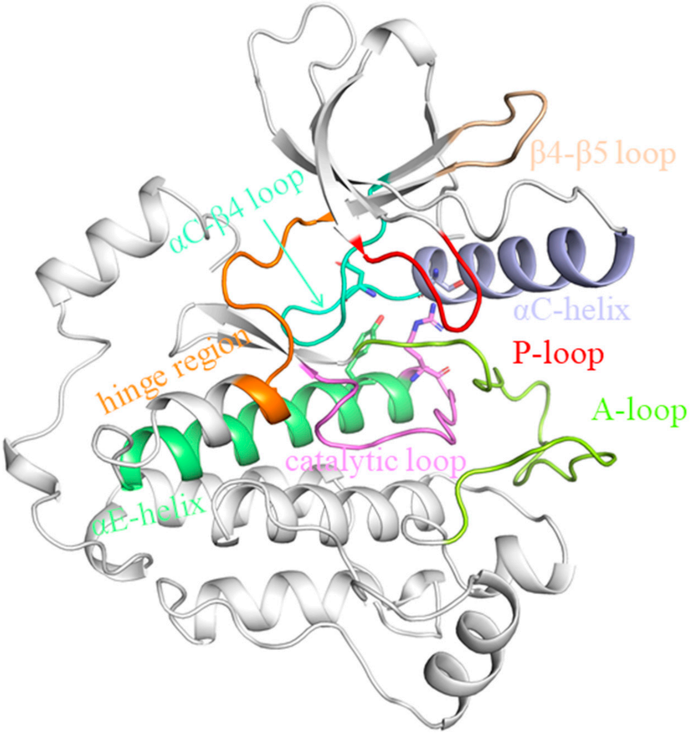

Figure S2. The structure of EGFR with important regions highlighted: the P-loop in red, the αC-helix in light blue, the αC-β4 loop in green cyan, the β4-β5 loop in wheat, the hinge region in orange, the αE-helix in lime, the catalytic loop in violet, and the A-loop in limon.

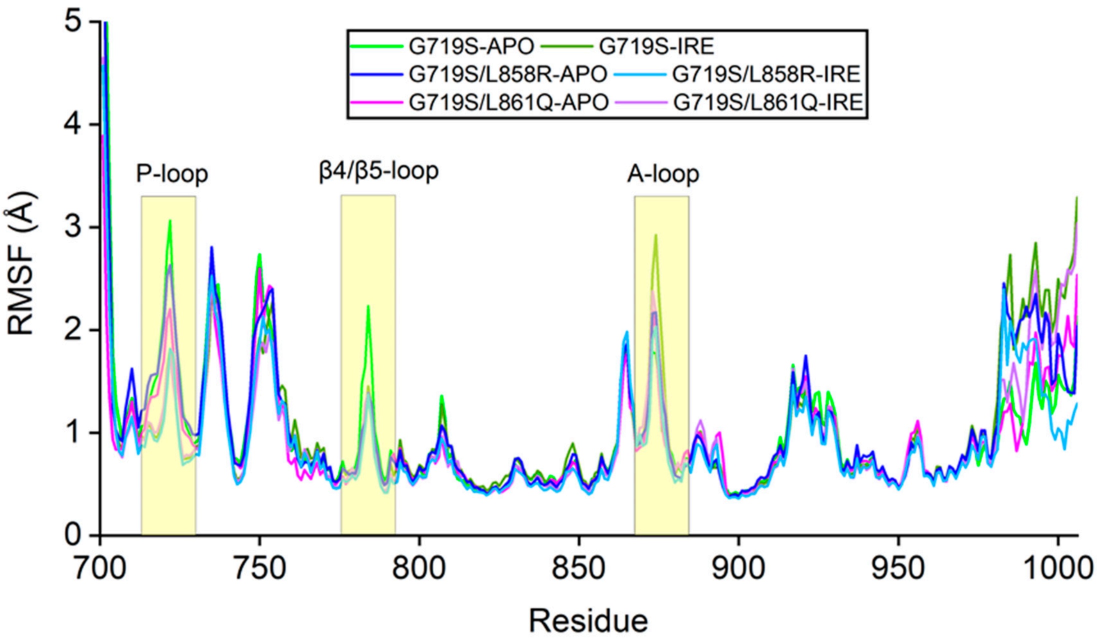

Figure S3. The flexibility of EGFRs during the simulations. The average RMSF of each residue in the last 200-ns MD trajectories using the average structure as the reference. Several important regions are highlighted.

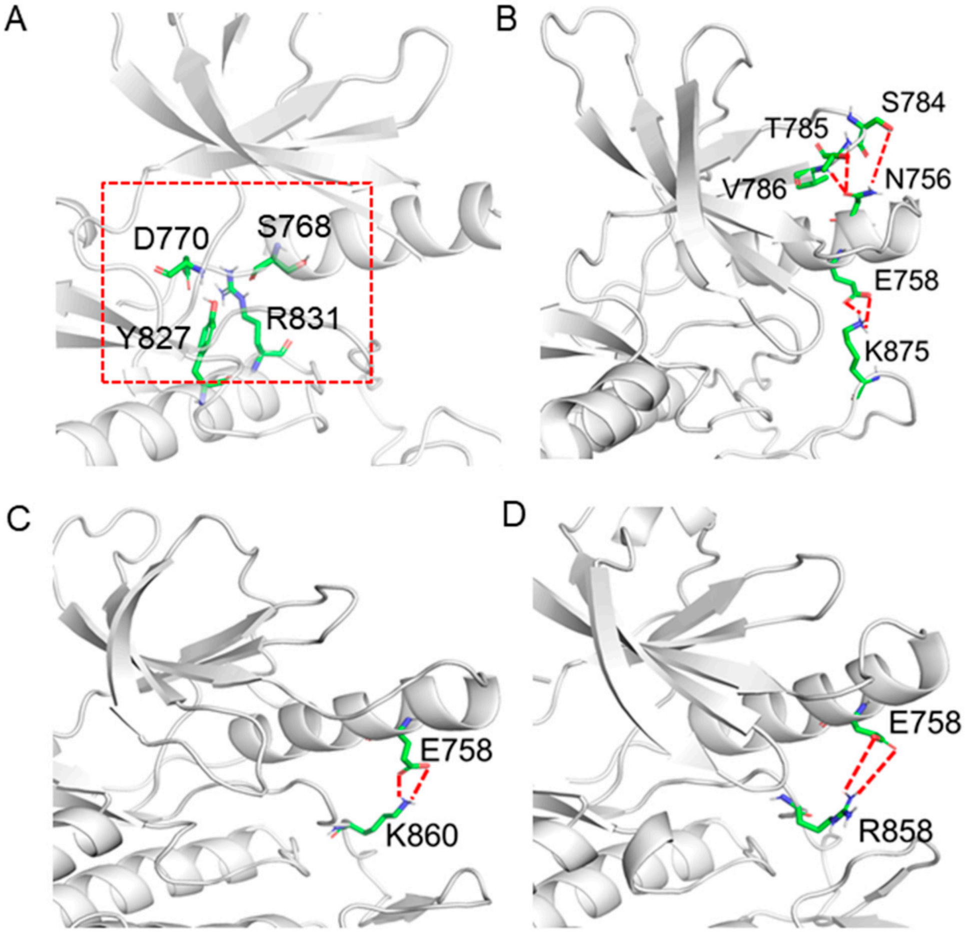

Figure S4. The hydrogen-bond interactions contribute to the stability of the αC-helix. (A) Hydrogen-bond interactions of S768, D770, Y827 and R831 residues in the EGFR kinase domain. (B) Side chains of residues S784, T785 and V786 in the β4-β5 loop make hydrogen bonds with that of N756 in the αC-helix. Side chain of K875 in the C terminal of the A-loop makes hydrogen bond with that of E758 in the αC-helix. (C) Side chain of K860 in the N-terminal of the A-loop makes hydrogen bond with that of E758 in the αC-helix. (D) Side chain of R858 in the N terminal of the A-loop makes hydrogen bond with that of E758 in the αC-helix.

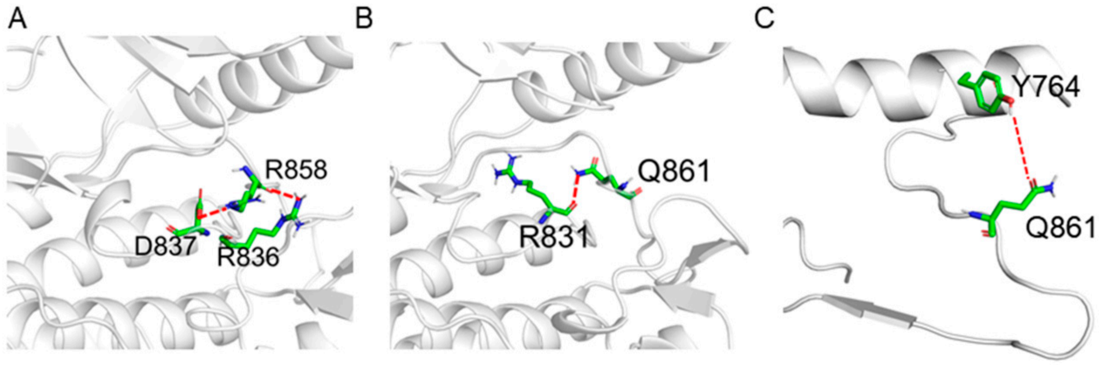

Figure S5. The hydrogen-bond interactions introduced by the co-occurring mutated residue. (A) Hydrogen bonds among the side chains of mutated R858 in the A-loop and R836/D837 in the catalytic loop. (B) Hydrogen bond between the side chains of mutated Q861 in the A loop and R831in the catalytic loop. (C) Hydrogen bond between the side chains of mutated Q861 in the A-loop and Y764 in the αC-helix.

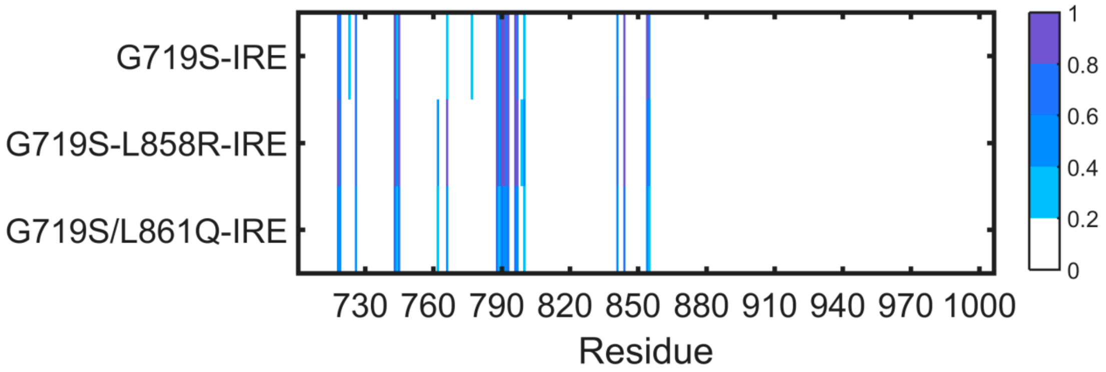

Figure S6. Contact map between IRE and EGFR in the three IRE-bound systems. The distance cutoff was set to 5 Å.

Table S1. The full names and abbreviations of twenty common amino acids.

Serial number	Full name	Abbreviation
1	Glycine	Gly/G
2	Alaine	Ala/A
3	Leucine	Leu/L
4	Isoleucine	Ile/I
5	Valine	Val/V
6	Proline	Pro/P
7	Phenylalanine	Phe/F
8	Methionine	Met/M
9	Tryptophan	Trp/W
10	Serine	Ser/S
11	Glutamine	Gln/Q
12	Threonine	Thr/T
13	Cysteine	Cys/C
14	Asparagine	Asn/N
15	Tyrosine	Tyr/Y
16	Aspartic acid	Asp/D
17	Glutamic acid	Glu/E
18	Lysine	Lys/K
19	Arginine	Arg/R
20	Histidine	His/H

Table S2. The full names and abbreviations of nouns in this work.

Serial number	Full name	Abbreviation
1	Gefitinib	IRE
2	Conventional molecular dynamic	CMD
3	Steered molecular dynamic	SMD
4	Principal component analysis	PCA
5	Root mean square deviation	RMSD
6	Root-mean-square fluctuation	RMSF
7	Radius of gyration	R_g
8	Solvent-accessible surface area	SASA
